# Genetic Variations in Inflammatory Response Genes and Their Association with the Risk of Prostate Cancer

**DOI:** 10.1155/2015/674039

**Published:** 2015-12-14

**Authors:** Xin Cui, Hao Yan, Tong-Wen Ou, Chun-Song Jia, Qi Wang, Jian-Jun Xu

**Affiliations:** Department of Urology, Xuanwu Hospital, Capital Medical University, Beijing 100053, China

## Abstract

Prostate cancer is a common cancer in men. Genetic variations in inflammatory response genes can potentially influence the risk of prostate cancer. We aimed to examine the association between *PPARG* Pro12Ala, *NFKB1* -94 ins/del, *NFKBIA* -826C/T, *COX-1* (50C>T), and *COX-2* (-1195G>A) polymorphisms on prostate cancer risk. The genotypes of the polymorphisms were ascertained in 543 prostate cancer patients and 753 controls through PCR-RFLP and the risk association was evaluated statistically using logistic regression analysis. The *NFKB1* -94 polymorphism was shown to decrease prostate cancer risk in both heterozygous and homozygous comparison models (odds ratios of 0.74 (95% CI = 0.58–0.96) (*P* = 0.02) and 0.57 (95% CI = 0.42–0.78) (*P* < 0.01), resp.). An opposite finding was observed for *COX-2* (-1195) polymorphism (odds ratios of 1.58 (95% CI = 1.15–2.18) (*P* < 0.01) for heterozygous comparison model and 2.08 (95% CI = 1.48–2.92) (*P* < 0.01) for homozygous comparison model). No association was observed for other polymorphisms. In conclusion, *NFKB1* -94 ins/del and *COX-2* (-1195G>A) polymorphisms may be, respectively, associated with decreased and increased prostate cancer risk in the Chinese population.

## 1. Introduction

Prostate cancer is a form of cancer in which malignant cells develop in the prostate gland of the male reproductive system. Prostate cancer is the second leading type of cancer in men globally, with an age-standardized incidence rate of 31.1 per 100,000 men [1]. In 2012 alone, a total of 1,111,689 cases of prostate cancers were diagnosed, and approximately 307,471 deaths were attended [1]. In comparison, the estimated numbers of incidence and mortality of the cancer were 679,000 and 221,000, respectively, in 2002 [2]. Thus, a marked increase in the incidence and mortality of prostate cancer has been observed during the past decade, and development of an effective screening strategy for identifying individuals at risk of prostate cancer is greatly needed. This is especially true for the Chinese population, in which the mortality-to-incidence rate ratio (MR/IR) has been consistently higher in comparison to the average MR/IR of Asian countries and Western populations [3, 4].

Established etiological factors of prostate cancer include age, ethnicity, diet, and environmental carcinogen exposure [5]. The interactions among the above elements are believed to play significant roles in contributing to prostate carcinogenesis, although the precise mechanisms of this have not been fully understood [5]. In addition, in recent years, chronic inflammation of the prostate and, importantly, genetic profile of an individual have been shown to play an equally important role in oncogenesis of the prostate [6, 7]. Epidemiological and molecular evidences implied that dysregulation of inflammatory response genes may promote the development and progression of approximately 20% of prostate malignancies by inducing DNA damage, cell proliferation enhancement, apoptosis inhibition, and angiogenesis simulation [8].

The most common cause of inflammatory response gene dysregulations is mutations in high penetrance genes [9]. Despite the fact that such mutations can strongly increase the risk of prostate cancer, they are only present in a small frequency in the general population [9]. In comparison, genetic variations in the form of single nucleotide polymorphisms (SNPs) in low penetrance genes can increase the risk of cancer modestly, but they are more prevalent in the general population and, thus, their overall impact could be substantial [10]. SNPs may alter the expression and functionality of the protein products [11], and many studies have showed a relationship between inherited variations in inflammatory response genes and the risk of prostate carcinogenesis [12–16]. However, the influence of several common SNPs in the inflammatory response genes, either individually or in combinations, on prostate cancer risk has been understudied in the Chinese population. This includes the* PPARG *Pro12Ala polymorphism,* NFKB1*-94 ins/del polymorphism,* NFKBIA*-826C/T polymorphism,* COX-1 *(50C>T) polymorphism, and* COX-2 *(-1195G>A) polymorphism. The present study aimed to investigate the association of the above polymorphisms with the risk of prostate cancer in the Chinese population.

## 2. Materials and Methods

### 2.1. Study Subjects

The study was approved by the Medical Ethics Committee of the Xuanwu Hospital, Beijing, China. All subjects were recruited from Xuanwu Hospital, Beijing, China, between October 2010 and August 2014. A total of 543 males who were diagnosed by qualified physicians as prostate cancer patients based on clinical, pathological, and laboratory tests were recruited into the study. To ensure consistency, only patients with high grade prostate cancer (Gleason score > 7) who had a PSA level of >10 ng/mL were included in the study. At the same period of time, a total of 753 healthy controls were enrolled into this study. Eligibility criteria for control included no individual history of cancer and no known genetic syndromes associated with prostate cancer and a PSA level of <2 ng/mL. Blood samples were collected from the subjects who meet the criteria mentioned above after obtaining written informed consent.

### 2.2. SNP Genotyping

Genomic DNA extraction was done by using EasyPure Blood Genomic DNA Kit (TransGen Biotech, Beijing, China) after the blood samples were taken from peripheral blood of all subjects. The DNA was used for genotyping of the five SNPs by using the PCR-RFLP method. All PCR were performed in a 25 *μ*L reaction mixture containing standard PCR buffer, 1.5 mM MgCl_2_, 0.25 mM dNTP, 1 unit Taq polymerase, and 0.4 *μ*M of each primer. For all polymorphisms, approximately 10% of the samples were chosen at random and sequenced to confirm the genotypes from the amplified PCR product. The laboratory personnel performing the genotyping were blinded to the identity, the case-control status, and source of the samples.

#### 2.2.1. *PPARG* Pro12Ala Polymorphism

The PCR primers used for amplification of the region containing the* PPARG *Pro12Ala polymorphism were 5′-GCC AAT TCA AGC CCA GTC-3′ and 5′-GAT ATG TTT GCA GAC AGT GTA TCA GTG AAG GAA TCG CTT TCC-3′. The annealing temperature of the reaction was 58°C. The PCR amplification produced a fragment of 270 bp in size, which was then digested using BstUI restriction enzyme. The fragment was cleaved into a 227 bp and a 43 bp fragment for the Ala/Ala genotype while the Pro/Pro genotype was uncut. Apart from that, heterozygous genotype (Pro/Ala) was detected by the presence of all the above bands in agarose gel.

#### 2.2.2. *NFKB1*-94 ins/del Polymorphism

The PCR for amplification of the region containing the* NFKB1*-94 ins/del polymorphism was performed by using 5′-TGG GCA CAA GTC GTT TAT GA-3′ and 5′-CTG GAG CCG GTA GGG AAG-3′ primers, which were annealed to the genomic DNA at 61°C. PflMI restriction enzyme was subsequently used to digest the amplified PCR product of 281 bp in size. For ins/ins genotype, the absence of restriction site resulted in the undigested fragment of 281 bp after the reaction. On the other hand, a 240 bp and a 45 bp fragment were obtained for the del/del genotype, while a 281 bp, a 240 bp, and a 45 bp fragment were found in the ins/del genotype.

#### 2.2.3. *NFKBIA*-826C/T Polymorphism

The* NFKBIA*-826C/T polymorphism was genotyped by first amplifying the region of interest by using the following PCR primers: 5′-GGT CCT TAA GGT CCA ATC G-3′ and 5′-GTT GTG GAT ACC TTG CAC TA-3′ at an annealing temperature of 59.5°C. This produced an amplicon of 200 bp in size. Restriction enzyme digestion was performed with BfaI restriction enzyme. The TT genotype was uncut by the enzyme and remained 200 bp, but the CC genotype was cleaved into 180 bp and 20 bp bands. Heterozygotes showed all three bands on agarose gel.

#### 2.2.4. *COX-1 *(50C>T) Polymorphism

The PCR primers used were 5′-GGT GCC CGG TGG GGA ATT TTC-3′ and 5′-GAG GGG AAA GGA GGG GGT TG-3. The annealing temperature used was 60°C. The PCR reaction generated a product of 245 bp. Then, the fragment was digested by using SmuI restriction enzyme. The TT genotype was not cut by the enzyme, hence remaining as a 245 bp band on agarose gels. The CC genotyped, on the other hand, was cleaved into two fragments of 120 bp and 125 bp in size. Heterozygotes showed the presence of all the above bands in agarose gel.

#### 2.2.5. *COX-2 *(-1195G>A) Polymorphism

The PCR primers used for amplification of the region containing the* COX-2* (1195G>A) polymorphism were 5′-CCC TGA GCA CTA CCC ATG AT-3′ and 5′-GCCTTCATAGGAGATACTGG-3′. The annealing temperature of the reaction was 62°C. The PCR amplification gave a product of 273 bp in size, which was then digested using PvuII restriction enzyme. The AA genotype was undigested, while the PCR product was cleaved into a 220 bp and a 53 bp fragment for the GG genotype. Apart from that, heterozygous genotype (AG) was detected by the presence of all the above bands on the agarose gel.

### 2.3. Statistical Analysis

Statistical analyses were carried out using the SPSS software package 18.0 (SPSS Inc., Chicago, IL). Mean age for cases and controls was evaluated by using Student's *t*-test. On the other hand, chi square test (*χ* ^2^ test) was used to determine the significance difference in the smoking status and the distribution of genotypes of the cases and controls. Odds ratios (ORs) and the corresponding 95% confidence intervals (CI) calculated by using logistic regression analysis were used to analyze the association between the polymorphic genotypes and prostate risk, based on heterozygous and homozygous comparison models, with the wild type genotype served as the reference. Odds ratios (ORs) of >1.00 indicated a positive risk association and vice versa. Interaction between the polymorphisms and smoking status, as well as between polymorphisms in related genes (*NFKB1* and* NFKBIA*;* COX-2* and* COX-1*), was tested by using the same logistic regression model as above, by using SPSS software. *P* values of <0.05 were significant. For analyses involving gene-gene and gene-environment interaction, Bonferroni correction was applied for multiple testing, in which case the statistical significance was assumed at *P* < 0.025.

## 3. Results

### 3.1. General Characteristics

Table 1 shows the difference between cases and controls in terms of age and smoking status. The ages of cases ranged from 48 to 87 years, and those of controls ranged from 46 to 85 years. The mean age was 69.90 ± 8.43 for cases while the mean age was 69.38 ± 8.76 for controls. No significant difference was observed between the mean ages of both groups (*P* = 0.29). For both cases and controls, the frequency of never smokers was slightly higher than ever smokers. In cases, 247 (45.49%) were ever smokers and 296 (54.51%) were never smokers, while, in controls, 329 (43.69%) were ever smokers and 424 (56.31%) were never smokers. The difference in the distribution of ever and never smokers among cases and controls was not significant (*P* = 0.52).

### 3.2. Distribution of Genotypes

The distribution of genotypes of the five polymorphisms in cases and controls is shown in Table 2. For the* NFKB1*-94 ins/del polymorphism, the frequency of ins/ins, ins/del, and del/del genotypes was 198, 246, and 99 in cases and 212, 355, and 186 in controls. Significant difference was observed between cases and controls in the distribution of genotypes (*χ* ^2^ = 13.12, *P* < 0.01).

Apart from that, another polymorphism which showed significant difference between cases and controls in the genotypic distribution was the* COX-2* (-1195G>A) polymorphism. Among the cases, 71, 269, and 203 subjects had GG, GA, and AA genotypes, respectively. On the other hand, the distribution of the genotypes among the controls was 158, 378, and 217, respectively (*χ* ^2^ = 18.34, *P* < 0.01).

For the three other polymorphisms, no statistically significant difference was observed between cases and controls in the genotypic distribution (*P* > 0.05). For* NFKBIA*-826C/T polymorphism, the distribution of the CC, CT, and TT genotypes was 382, 152, and 9 in cases and 541, 198, and 14 in controls (*χ* ^2^ = 0.51, *P* = 0.77). For* COX-1* (50C>T) polymorphism, the CC, CT, and TT genotypes were, respectively, present in 452, 83, and 8 cases as well as 655, 92, and 6 controls (*χ* ^2^ = 4.05, *P* = 0.13). On the other hand, for* PPARG Pro12Ala* polymorphism, 483, 57, and 3 cases had Pro/Pro, Pro/Ala, and Ala/Ala genotypes, while the distribution of the same genotypes in controls was 676, 73, and 4, respectively (*χ* ^2^ = 0.23, *P* = 0.89).

For all polymorphisms, the genotype distribution did not deviate significantly from Hardy Weinberg equilibrium, both among cases and among controls (*P* > 0.05).

### 3.3. Risk Association: Overall

Table 3 shows the association between the five polymorphisms and prostate cancer risk in the population studied. For* NFKB1*-94 ins/del polymorphism, a significantly decreased prostate cancer risk was observed in the heterozygous comparison model (ins/del versus ins/ins) and homozygous comparison model (del/del versus ins/ins). In heterozygous comparison model, the odds ratio was 0.74 (95% CI = 0.58–0.96), with a *P* value of 0.02. On the other hand, in homozygous comparison model, the odds ratio was 0.57 (95% CI = 0.42–0.78, *P* < 0.01).

For* COX-2 *(-1195G>A) polymorphism, a significantly increased prostate cancer risk was observed in the heterozygous comparison model (GA versus GG) and homozygous comparison model (AA versus GG). In heterozygous comparison model, the odds ratio was 1.58 (95% CI = 1.15–2.18), with a *P* value of 0.01. On the other hand, in homozygous comparison model, the odds ratio was 2.08 (95% CI = 1.48–2.92, *P* < 0.01).

For* NFKBIA*-826C/T polymorphism, statistically not significant risk association was observed. The heterozygous comparison model (CT versus CC) showed an odds ratio of 1.09 (95% CI = 0.85–1.39) and a *P* value of 0.51. On contrary, the homozygous comparison model (TT versus CC) showed an odds ratio of 0.9104 (95% Cl = 0.39–2.13) and a *P* value of 0.83.

Similarly, lack of significant association was observed for* COX-1 *(50C>T) polymorphism and* PPARG *Pro12Ala polymorphism. Both the polymorphisms showed positive odds ratio values for both heterozygous and homozygous comparison models. For* COX-1 *(50C>T) polymorphism, the odds ratio obtained in the heterozygous comparison model was 1.31 (95% CI = 0.95–1.80, *P* = 0.10), while that in the homozygous comparison model was 1.93 (95% CI = 0.67–5.61, *P* = 0.23). On the other hand, for* PPARG *Pro12Ala polymorphism, the odds ratio was 1.09 (95% CI = 0.76–1.58) in the heterozygous comparison model (*P* = 0.63) and 1.05 (95% CI = 0.23–54.71) in the homozygous comparison model (*P* = 0.95).

### 3.4. Risk Association: Interaction of Polymorphisms with Smoking Status

The interaction between smoking status and the polymorphisms in modulating prostate cancer risk was also formally investigated, and the results are shown in Table 4. Significant interaction with smoking status was observed only for the* COX-2 *(-1195G>A) polymorphism (*P* < 0.01), in which a significant risk association was observed between the AA genotype and ever smokers (OR = 1.76, 95% CI = 1.28–2.41, *P* < 0.01).

### 3.5. Risk Association: Gene-Gene Interactions

The effect of interactions between polymorphisms in related genes (*NFKB1* and* NFKBIA*;* COX-2* and* COX-1*) on the risk of prostate cancer was also examined (Table 5). Both* NFKB1* and* NFKBIA* combination and* COX-2* and* COX-1* combination showed statistically significant interactions (*P* = 0.03 and *P* = 0.02, resp.). For the interactions between* COX-2*-1195G>A and* COX-1 *(50C>T) polymorphisms, interaction between* COX-2* (-1195AA) *∗ COX-1 *(50CT) genotypes conferred a risk increment with OR = 1.83 (95% CI = 1.06–3.17, *P* = 0.03). However, this was deemed not statistically significant after application of Bonferroni correction, where *P* < 0.025 was considered as significant. For the interactions between* NFKB1*-94 ins/del and* NFKBIA*-826C/T polymorphisms, statistically significant prostate cancer risk association was not observed for any polymorphic combination investigated. No other genotypic interactions yielded a statistically significant association.

## 4. Discussion

In this study, we investigated and established the association of five inflammation-related genetic polymorphisms, namely,* PPARG *Pro12Ala,* NFKB1*-94 ins/del,* NFKBIA*-826C/T,* COX-1 *(50C>T), and* COX-2 *(-1195G>A) polymorphisms, with prostate cancer risk in a Chinese population. These polymorphisms have been widely investigated for their associations with other cancers or in other populations, but report is scarce or unavailable for prostate cancer in the Chinese population. This novelty of data represents one of the strengths of the present study. Additionally, our study involved a considerably large number of subjects, which increases the reliability of the results obtained. Apart from that, our study analyzed the combined effect of polymorphisms in a similar pathway (i.e.,* NFKB1* and* NFKBIA*;* COX-2* and* COX-1*), an approach which has been neglected in many previous reports.

*PPARG *gene encodes for peroxisome proliferator-activated receptor-*γ* (PPAR-*γ*), which is a ligand-activated transcription factor that is pleiotropic in nature. PPAR-*γ* is best known for its function in the regulation of adipogenesis and fatty acid metabolism, but it appears that the protein also plays a role in several carcinogenic processes, including apoptosis, cell cycle control, and, notably, inflammation [17]. The Pro12Ala polymorphism has been shown previously to affect the binding affinity of PPAR-*γ* towards its target molecules [18]. Therefore, we postulated that this polymorphism could influence the risk of prostate cancer. However, we observed a lack of association between the* PPARG *Pro12Ala polymorphism and prostate cancer risk in our population. We propose that this observation could be due to the fact that although different genotypes of the polymorphism have different binding affinity to its target molecules, the difference is subtle and does not influence the ability of PPAR-*γ* in activating the transcription of its target molecules. Our result was in agreement with a study from Finland [19] and in partial agreement with another study from USA [20], which showed that the polymorphism was associated with an increased prostate cancer risk only among individuals with high body mass index.

On the other hand,* NFKB1* and* NFKBIA* encode for NF-*κ*B1 and its inhibitor, I*κ*B*α*, respectively. Both NF-*κ*B1 and I*κ*B*α* are part of the NF-*κ*B pathway, which plays an important role in inflammation and other carcinogenic processes. The -94 ins/del polymorphism of* NFKB1* and -826C/T polymorphism of* NFKBIA* have been shown to affect the transcriptional activity of the respective genes, as the polymorphisms were located in the promoter region of the genes [10, 12]. We observed that the* NFKB1*-94 ins/del polymorphism was associated with decreased risk of prostate cancer in both heterozygous and homozygous comparison models, suggesting that the variant del allele may be linked to a reduced inflammatory status among its carriers and, therefore, a reduced risk. This risk reduction was in agreement with an in vitro assay which showed that the del allele decreased the transcriptional activity of NFKB1, thereby truncating its role in promoting inflammation, which in turn led to the decreased prostate cancer risk [21]. Our finding was also in agreement with two other previous studies on prostate cancers, namely, Zhang et al. [22] in China and Kopp et al. [13] in Denmark. For* NFKBIA* -826C/T polymorphism, no significant association with prostate cancer risk was observed. Despite many previous studies reported on the association (or lack of association) between the* NFKBIA* polymorphism and risk of various cancers [10, 23–25], we believe that this is the first work which examined its association with prostate cancer risk.

Cyclooxygenases are enzymes which catalyze the conversion of arachidonic acid to prostaglandins, which promote and regulate the process of inflammation. There are two isoforms of cyclooxygenases, namely, the constitutively expressed cyclooxygenase-1 and inducibly expressed cyclooxygenase-2, which are encoded by* COX-1* and* COX-2* genes, respectively. The* COX-1 *(50C>T) polymorphism is located within the coding region of the gene and can potentially influence the functional importance of the enzyme produced [26]. On the other hand, the promoter* COX-2 *(-1195G>A) polymorphism can alter the transcriptional activity of the gene [27]. Our result showed the absence of prostate cancer risk association for the* COX-1 *(50C>T) polymorphism. This indicates that, despite its functional importance, the polymorphism is not sufficiently strong to result in significant risk alteration among its carriers. We also showed the presence of an association between* COX-2 *(-1195G>A) polymorphism and increased prostate cancer risk in both heterozygous and homozygous comparison models. This suggests that the variant A allele of the* COX-2 *polymorphism could contribute to an increased prostate cancer risk among its carriers. The observation that the A allele increases prostate cancer risk can be explained by the fact that the A allele creates a c-Myb transcription factor binding site in the gene, resulting in an increased transcription of* COX-2 *[27]. This in turn leads to the greater promotion of inflammation, which mediates the process of carcinogenesis. Our study is the first to evaluate the association between* COX-1 *(50C>T) polymorphism and prostate cancer risk, whereas several previous reports were available for the association between* COX-2 *(-1195G>A) polymorphism and prostate cancer risk. Our study results were in agreement with Sugie et al. [28] in a Japanese population, but Wu et al. (Taiwan) [29], Cheng et al. (USA) [30], and Kopp et al. (Denmark) [13] showed the absence of risk association between* COX-2 *(-1195G>A) polymorphism and prostate cancer risk.

In summary, we have demonstrated the presence of a positive prostate cancer risk association for* COX-2 *(-1195G>A) polymorphism, a negative risk association for* NFKB1*-94 ins/del polymorphism, and absence of association for* PPARG* Pro12Ala polymorphism,* NFKBIA*-826C/T polymorphism, and* COX-1 *(50C>T) polymorphism. There are several limitations in this study. First, although the overall sample size was large, the sample size became small when analysis was performed separately between smokers and nonsmokers and when the genotypes were combined. In addition, we only included five polymorphisms which were considered more well established based on our literature review. Therefore, a more extensive examination for a greater number of polymorphisms in a larger sample size is needed and recommended for future work for reconfirmation of our findings.

## Figures and Tables

**Table 1:** Difference between cases and controls characteristics.

Characteristics	Case (*n* = 543)	Control (*n* = 753)	*P*
Age			0.29
Mean ± SD	69.90 ± 8.43	69.38 ± 8.76	
Range	48–87	46–85	
Smoking status			0.52
Ever smokers	247	329	
Never smokers	296	424	

**Table 2:** Distribution of genotypes.

Genotype	Case (*n* = 543)	Control (*n* = 753)	*χ* ^2^	*P*
*NFKB1* -94 Ins/del			13.12	<0.01
Ins/Ins	198	212		
Ins/Del	246	355		
Del/Del	99	186		
*NFKBIA* -826C/T			0.51	0.77
CC	382	541		
CT	152	198		
TT	9	14		
*COX-2* (-1195G>A)			18.34	<0.01
GG	71	158		
GA	269	378		
AA	203	217		
*COX-1* (50C>T)			4.05	0.13
CC	452	655		
CT	83	92		
TT	8	6		
*PPARG* Pro12Ala			0.23	0.89
Pro/Pro	483	676		
Pro/Ala	57	73		
Ala/Ala	3	4		

**Table 3:** Association between polymorphisms and prostate cancer risk.

Genotype	Case (*n* = 543)	Control (*n* = 753)	OR (95% CI)	*P*
*NFKB1* -94 ins/del				
Ins/Ins	198	212	Ref.	—
Ins/Del	**246**	**355**	**0.74 (0.58 to 0.96)**	**0.02**
Del/Del	**99**	**186**	**0.57 (0.42 to 0.78)**	**<0.01**
*NFKBIA* -826C/T				
CC	382	541	Ref.	—
CT	152	198	1.09 (0.85 to 1.39)	0.51
TT	9	14	0.91 (0.39 to 2.13)	0.83
*COX-2* (-1195G>A)				
** **GG	71	158	Ref.	—
GA	**269**	**378**	**1.58 (1.15 to 2.18)**	**0.01**
AA	**203**	**217**	**2.08 (1.48 to 2.92)**	**<0.01**
*COX-1* (50C>T)				
CC	452	655	Ref.	—
CT	83	92	1.31 (0.95 to 1.80)	0.10
TT	8	6	1.93 (0.67 to 5.61)	0.23
*PPARG* Pro12Ala				
Pro/pro	483	676	Ref.	—
Pro/ala	57	73	1.09 (0.76 to 1.58)	0.63
Ala/ala	3	4	1.05 (0.23 to 4.71)	0.95

**Table 4:** Gene-environment interaction between the polymorphisms and smoking status.

Interaction	*P* for interaction	OR (95% CI)	*P*
*NFKB1* *∗* smoking status	0.31		
*NFKB1* -94 ins/ins *∗* nonsmokers		Ref.	—
*NFKB1* -94 ins/del *∗* smokers		1.01 (0.76–1.33)	0.96
*NFKB1* -94 del/del *∗* smokers		0.74 (0.50–1.10)	0.14

*NFKBIA* *∗* smoking status	0.71		
*NFKBIA* -826CC *∗* nonsmokers		Ref.	—
*NFKBIA* -826CT *∗* smokers		1.13 (0.80–1.60)	0.49
*NFKBIA* -826TT *∗* smokers		1.41 (0.35–5.66)	0.63

*COX-2* *∗* smoking status	**<0.01**		
*COX-2* (-1195GG) *∗* nonsmokers		Ref.	—
*COX-2* (-1195GA) *∗* smokers		1.29 (0.98–1.69)	0.07
*COX-2* (-1195AA) *∗* smokers		**1.76 (1.28–2.41)**	**<0.01**

*COX-1* *∗* smoking status	0.13		
*COX-1* (50CC) *∗* nonsmokers		Ref.	—
*COX-1* (50CT) *∗* smokers		1.46 (0.92–2.32)	0.11
*COX-1* (50TT) *∗* smokers		2.38 (0.57–10.00)	0.24

*PPARG* *∗* smoking status	0.23		
*PPARG* Pro/Pro *∗* nonsmokers		Ref.	—
*PPARG* Pro/Ala *∗* smokers		0.92 (0.50–1.68)	0.11
*PPARG* Ala/Ala *∗* smokers		N/A	N/A

There were two factors involved (polymorphism and smoking status) in each testing; hence, significance is assumed at *P* < 0.025 after application of Bonferroni correction.

**Table 5:** Combination of polymorphisms and prostate cancer risk.

Interaction	*P* for interaction	OR (95% CI)	*P*
*NFKB1* *∗* *NFKBIA* polymorphisms	0.03		
*NFKB1* -94 ins/ins *∗* *NFKBIA* -826CC		Ref.	—
*NFKB1* -94 ins/del *∗* *NFKBIA* -826CT		0.99 (0.71–1.38)	0.94
*NFKB1* -94 del/del *∗* *NFKBIA* -826TT		3.16 (0.81–12.28)	0.10
*NFKB1* -94 ins/del *∗* *NFKBIA* -826CT		0.65 (0.39–1.07)	0.09
*NFKB1* -94 del/del *∗* *NFKBIA* -826TT		N/A	—

*COX-2* (-1195GG) *∗* *COX-1* (50CC)	0.02	Ref.	—
*COX-2* (-1195GA) *∗* *COX-1* (50CT)		1.34 (0.87–2.07)	0.19
*COX-2* (-1195AA) *∗* *COX-1* (50CT)		1.83 (1.06–3.17)	0.03
*COX-2* (-1195GA) *∗* *COX-1* (50TT)		N/A	—
*COX-2* (-1195AA) *∗* *COX-1* (50TT)		1.47 (0.30–7.29)	0.64

There were two polymorphisms involved in each testing; hence, significance is assumed at *P* < 0.025 after application of Bonferroni correction.
